# Interactive Self-Reporting as Behavioural Cue Elicitor for Online-Classroom Assessment

**DOI:** 10.3390/bs12070232

**Published:** 2022-07-14

**Authors:** Hao Xu

**Affiliations:** National Research Centre for Foreign Language Education, Beijing Foreign Studies University, Beijing 100089, China; xuhaokent@bfsu.edu.cn

**Keywords:** interactive self-reporting, intensity of interaction, behavioural cues, classroom assessment, online teaching, assessment data

## Abstract

How teachers should elicit and draw on behavioural cues for online-classroom assessment is of much interest to both researchers and practising teachers. Aiming to explore how to enhance interactive self-reporting as a behavioural cue elicitor for online-classroom assessment, this study adopted a large-scale questionnaire survey to investigate the effects of the intensity of interaction in interactive self-reporting and teachers’ professional experience on the quality of assessment data in online teaching. Results showed that only the intensity of teacher’s follow-up interaction regarding interactive self-reporting had a significant impact on the quality of the assessment data. Specifically, as a behavioural cue elicitor, interactive self-reporting may be best utilised when interaction of a moderate intensity is employed by the teacher following students’ self-report. The accuracy and efficiency of interactive self-reporting as a means to obtain assessment data in online teaching can only, thus, be best synergised. Otherwise, the accuracy and efficiency may be reduced to a wax-and-wane relationship, i.e., each one increases at the expense of the other.

## 1. Introduction

Classroom assessment is a key component for effective teaching and learning, as it provides important information for both teachers and students to identify and analyse the learning difficulties and, thus, for teachers to seek related pedagogical intervention or for students to engage in self-directed efforts to tackle their difficulties [1]. The role of classroom assessment as a crucial part of teaching and learning is equally, if not more, important in online teaching and learning. Although online learning, owing to technical and contextual factors, entails a range of behaviours, which are different from those in offline learning [2], classroom assessment is still needed as an indispensable part of teaching and learning to examine students’ behaviours and evaluate their learning outcomes [3]. Online assessment may even develop from a novel method of evaluation to a norm, as an increasing number of courses have been making online assessment mandatory. [4] Therefore, more research endeavours are anticipated, to explore how classroom assessment should be effectively conducted for online teaching and learning, which involves different learning behaviours from those in offline teaching and learning. Specifically, what kind of behavioural cues should teachers draw on for online-classroom assessment should be of primary interest to both researchers and practising teachers. Whilst behavioural cues have long been investigated as an important source of effective assessment, relatively little research has examined how they may impact online assessment and how their impact may differ for online assessment from that of offline assessment [5].

Although a variety of sources can be drawn on to elicit behavioural cues for the purpose of classroom assessment [6], behavioural cues are mostly obtained via teachers’ direct observation, particularly in an offline classroom [7]. Consequently, in online teaching, lack of access to such observation, which is not only direct but also multi-dimensional, may require teachers to seek alternative methods of obtaining students’ behavioural cues. A natural and effective approach to obtaining behavioural cues is for students to report their own learning process and outcome as an indirect lens for teachers to observe their behaviours [8,9]. Previous research has also shown the usefulness of students’ own reporting, for instance in the form of student feedback, in providing the teacher with important information with which to conduct assessment [10]. In this study, such an approach is conceptualised as self-reporting, which serves as a source of assessment data in online teaching and learning.

Due to factors such as selective attention and mistaken perception, students’ self-reporting may not accurately represent their learning behaviours, making teachers’ follow-up examination on such self-reporting a necessary step to ensure or enhance the accuracy of the assessment data from this source. In other words, teachers need to engage in a certain follow-up interaction as a kind of assessment-data enhancer. Such an enhancer should help teachers validate students’ self-reporting and, when necessary, elicit additional information or evidence that the self-reporting fails to include and present. Hence, the combination of a student’s self-reporting and a teacher’s follow-up interaction can be conceptualised as *interactive self-reporting*, consisting of the original student’s report, the teacher’s follow-up interaction as an assessment-data enhancer, and the student’s response to the enhancer. It seems most likely that interactive self-reporting can be utilised as an alternative means to elicit behavioural cues in online-classroom assessment.

However, teachers may need to properly regulate their follow-up interaction, so as to enhance the assessment data, and at the same time *not* to bring about concomitant negative effects such as provoking learner anxiety and taking too much time [11]. In other words, teachers’ follow-up interaction needs to be of moderate intensity. Therefore, the effects of the intensity of interaction on the quality of the assessment data obtained via interactive self-reporting in online teaching need to be more closely examined. The paucity of research so far on this issue has obviously created a research gap for us to fill.

This article, thus, reports on the results of a preliminary study that aims to answer the following research question:

Do interactions of different levels of intensity have different effects on the quality of assessment data in online teaching?

## 2. Methods

In this section, the research design will first be introduced, particularly how variables were designed. Then, specifics of the study will be elaborated, including research participants, instruments, procedures, and data analysis.

### 2.1. Design

This study primarily examines the effects of intensity of a teacher’s follow-up interaction on the quality of online-assessment data. Intensity of interaction (thereafter abbreviated as “IoI”) is, thus, an independent variable. Three levels of this independent variable are designed: (1) no interaction, i.e., the teacher does not interact with the student upon receipt of the student’s self-reporting; (2) brief interaction, i.e., after the student completes self-reporting, the teacher asks only one question, which the student responds to without the teacher’s further response; and (3) elaborate interaction, i.e., the teacher and the student engage in two rounds of question and answer after the student’s self-reporting. In other words, in the elaborate interaction, the teacher and the student engage in one more round of question and answer than in the brief interaction. In this study, each research participant would be exposed to scenarios for all of the three levels, making IoI a within-group variable.

Since teachers who are in different stages of professional development may respond differently to scenarios for different levels of IoI, the study also examines professional experience (thereafter as “PE”) as a between-group independent variable, consisting of two levels, i.e., novice teachers (with 1–3 years of teaching experience) and experienced teachers (with 10–15 years of teaching experience) [12].

The quality of the assessment data (hereafter referred to as “ADQ”), the dependent variable of the study, is investigated via research participants’ individual judgments about how well the assessment data can inform a teacher about a student’s learning difficulties. In other words, the ADQ in this study is the perceived quality as reported by the research participants. This may be a limitation of the current study, and, thus, future studies may more directly investigate the ADQ, with reference to how accurately the assessment data reflects students’ learning gains and difficulties. In sum, this study adopts a 3 × 2 mixed design to address the research question.

### 2.2. Participants

Research participants were recruited via Wechat groups, a social media platform widely used in China. The advertisement that invited junior-high-school teachers of English as a Foreign Language (EFL) to participate was posted in 49 Wechat groups, with a total of approximately 17,000 members who were all EFL school teachers. The group members were also encouraged to share the advertisement with anyone who might be interested. The link to the questionnaire survey was attached to the advertisement.

When the research participants started to view and respond to the questionnaire survey, they would first see a page stating the purpose of the study and their rights as participants. They would be able to proceed with the survey, after they formally consented to participating in the study.

Altogether, 3967 junior high school teachers responded to the questionnaire survey, among whom 2235 teachers competed the survey, i.e., they responded to all of the required questions. There were 219 novice teachers who had 1–3 years of teaching experience and 1789 experienced teachers who had 10–15 years of teaching experience that completed the questionnaire survey. The 219 experienced teachers were then randomly selected as the experienced-teacher group, the size of which was, thus, made comparable to the novice-teacher group. To summarise, the participants of this study consisted of 438 EFL teachers from junior high schools in China, including a novice-teacher group (PE: *M* = 3.059 years) and an experienced-teacher group (PE: *M* = 12.333 years).

### 2.3. Instruments and Procedures

The instruments of the study consisted of three videotaped online-teaching scenarios and an ADQ-judgment scale. All of the three scenarios were grammar-teaching episodes that focused, respectively, on the past perfect tense (had done), the future perfect tense (will have done), and the future in the past tense (would do). In each three-phase scenario, which is a short video showing the same female teacher teaching the same five female students in an online class, the teacher first presented the rule of the grammar item in focus, explaining the usage and form using three examples (this **presentation phase** took 3.5 min). Then, the teacher asked the students to do a fill-in-the-blank exercise containing 10 sentences such as “Johnson suddenly realised that he __________ (forget) his mother’s birthday.” After the **exercise phase**, which took 2.5 min (only showing how the teacher assigned the exercise and checked the answers), the teacher initiated the **assessment phase**, showed on the screen a new sentence with a blank, and asked the same student (in all three scenarios) to fill in the blank and report how well she thought she had mastered the grammar item. The interactive self-reporting transcripts for each of the three scenarios are shown as follows:

**No interaction** (the past perfect tense)

Sentence: Before he went to school, Jimmy __________ (know) many English words.

Student: “The answer is ‘has known.’ *Wo zhidao zheli yinggai yong wanchengshi*. [said in Chinese, meaning ‘I know I should use the perfect tense here.’]”

Teacher: No response.

**Brief interaction** (the future perfect tense)

Sentence: By this month next year, Lily __________ (graduate) from the university.

Student: “The answer is ‘will graduate.’ *Wo zhidao zheli yinggai yong jianglaishi.* [I know I should use the future tense here.]”

Teacher: “That’s right. *Danshi, zheli you yige shijian zhuangyu. Ta shi shenme yisi?* [But there is a time adverbial. What does it mean?]”

Student: “Oh, I see. Then the answer should be ‘will have graduated.’”

**Elaborate interaction** (the future-in-the-past tense)

Sentence: Kent always thought he __________ (become) a pilot when he grew up.

Student: “The answer is ‘will become.’ *Wo zhidao zheli yinggai yong jianglaishi.* [I know I should use the future tense here.]”

Teacher: “That’s right. *Danshi, zhege juzi zhengtishang shi shenme shitai?* [But what is the tense of the whole sentence?]”

Student: “Oh, I see. Then the answer should be ‘would become.’”

Teacher: “Great! *Zhege juzi zenme gai jiu keyi yong* ‘will become’ *le*? [How can we revise the sentence so that ‘will become’ will be the correct answer?]”

Student: “Kent always thinks he will become a pilot when he grows up.”

After watching each of the three scenarios, the participants were asked to respond to the ADQ-judgment scale, a seven-point Likert scale that consisted of the following two items:

To what extent do you think the assessment data the teacher obtained accurately reflect the students’ learning?
To what extent do you think the assessment is efficiently conducted, supposedly in a real online class?
(select a number from 1–7, with 1 representing extremely small and 7 representing extremely large)

As is shown above, one item elicited the participants’ judgment on the accuracy of the assessment data obtained from interactive self-reporting for assessing the students’ learning outcome and identifying possible learning difficulties; the other item elicited the participants’ judgment on the efficiency of interactive self-reporting, as in a real online-classroom setting. Such an approach has been widely acknowledged as a valid means to collect data that pertain to judgement about or attitude towards different facets of the same construct.

After the participants gave formal consent to their participation in the study, they would be directed to a website, first showing the directions for the questionnaire survey. Then, each of them would be shown the three videotaped scenarios (approximately 7 min each) in a randomised order. After they viewed each scenario, they first responded to the ADQ-judgment scale, and then began viewing the next scenario. Each of the participants submitted six scores (1–7) in all. The purpose of adopting a 7-point-scale format instead of a 5-point format was to more accurately reflect the subtle difference the teacher participants might perceive between different scenarios.

### 2.4. Data Analysis

The mean score for each participants’ response (two scores, respectively, for accuracy and efficiency) to each of the three scenarios was calculated as the ADQ score for that scenario. Then, the analysis of variance (repeated measures), suitable for analysis involving one or more within-group factors, was conducted to determine the effects of IoI and PE, as well as their interaction, on the ADQ of interactive self-reporting.

## 3. Results

Descriptive statistics for the two groups’ accuracy and efficiency ratings as well as the ADQ scores are shown in Table 1 below:

As is demonstrated above, the IoI seemed to have an obvious impact on the participants’ judgment about the ADQ of the assessment practices in the three scenarios. Specifically, brief interaction received the highest ADQ score (*M* = 4.43), compared to the ADQ scores for no interaction (*M* = 4.18) and elaborate interaction (*M* = 4.17). Even when the accuracy and efficiency ratings are observed separately, brief interaction still outscored no interaction and elaborate interaction in both ratings. Interestingly, no interaction and elaborate interaction seemed to be characterised by a balance between accuracy and efficiency, i.e., when accuracy increased, efficiency seemed to decline, and vice versa. This might be the reason why the ADQ scores for no interaction and elaborate interaction seemed to be similar: accuracy and efficiency seemed to be achieved at the expense of each other.

Teachers’ PE, however, did not seem to demonstrate a strong impact on the ADQ scores, except for a seemingly slight difference for elaborate interaction between novice teachers (*M* = 4.24) and experienced teachers (*M* = 4.09). Experienced teachers seemed to think less favourably about elaborate interaction, particularly with regard to its efficiency.

To further determine the effects of IoI and PE on the ADQ, the analysis of variance (repeated measures) was conducted. The results showed that: (1) IoI significantly impacted the ADQ (*F* = 9.711, *p* = 0.000); (2) PE did not demonstrate a statistically significant impact on the ADQ (*F* = 1.114, *p* = 0.292); and (3) the interaction between IoI and PE did not significantly impact the ADQ (*F* = 0.819, *p* = 0.441). To further determine how the three levels of IoI impacted the ADQ, pairwise comparisons were made. Results showed that brief interaction significantly outscored no interaction (*p* = 0.001) and elaborate interaction (*p* = 0.000), while no interaction and elaborate interaction did not demonstrate a significant difference (*p* = 1.000).

Two more rounds of analysis of variance (repeated measures) were then conducted to more specifically determine the effects of IoI and PE on the accuracy and efficiency ratings. The results showed that: (1) IoI significantly impacted both the accuracy rating (*F* = 105.722, *p* = 0.000) and the efficiency rating (*F* = 99.765, *p* = 0.000); (2) PE did not demonstrate a statistically significant impact on accuracy (*F* = 1.168, *p* = 0.280) or efficiency (*F* = 0.157, *p* = 0.692); and (3) the interaction between IoI and PE did not significantly impact either accuracy (*F* = 0.229, *p* = 0.790) or efficiency (*F* = 2.591, *p* = 0.078). To further determine how the three levels of IoI impacted accuracy and efficiency, pairwise comparisons were made. Results showed that the accuracy rating increased with the IoI, i.e., brief interaction significantly outscored no interaction (*p* = 0.000), and elaborate interaction significantly outscored brief interaction (*p* = 0.017). However, the results also showed that the efficiency rating decreased with the IoI, i.e., no interaction significantly outscored brief interaction (*p* = 0.000), and brief interaction significantly outscored elaborate interaction (*p* = 0.000). This confirmed the wax-and-wane relationship between accuracy and efficiency within the ADQ well.

## 4. Discussion

So far, the effects of IoI and PE on the ADQ have been examined based on the dataset obtained via a questionnaire survey, which included three videotaped scenarios for online-classroom assessment. In response to the research question raised earlier, it can be concluded that in online teaching, the intensity of teachers’ follow-up interactions for interactive self-reporting has a significant impact on the quality of assessment data. As a behavioural cue elicitor, interactive self-reporting may be best utilised when brief interaction, rather than no interaction or elaborate interaction, is employed by the teacher following students’ self-reporting. In other words, the accuracy and efficiency of interactive self-reporting as a means to obtain assessment data in online teaching can be best synergised when brief interaction is employed. Otherwise, the accuracy and efficiency may be reduced to a wax-and-wane relationship, i.e., each one increases at the expense of the other.

As a means to elicit behavioural cues for online-classroom assessment, interactive self-reporting serves as a kind of dual mediation that enhances both the availability and the quality of assessment data. Students’ self-reporting enables the teacher to obtain assessment data that otherwise may not be as easily accessible as in an offline classroom. While students’ self-assessment has been widely acknowledged as an important source of assessment data in online teaching [8,10], previous studies have rarely investigated how the quality of self-reporting assessment data can be further enhanced via teachers’ follow-up interaction as a kind of intervention. This should be where the current study makes its unique contribution to the field of online-classroom assessment. Teachers’ follow-up interaction, as has been demonstrated by the results of this study, needs to be regulated to a moderate intensity, so as to achieve a balance point between the gain in accuracy and loss in efficiency. Although some previous studies have mentioned the relationship between accuracy and efficiency in testing and assessment [13], they have not directly articulated such an interactive relationship or attempted to measure this relationship by empirical research. In sum, the current study contributes to our understanding of how teachers perceive and utilise students’ self-reporting, as an important source of online-assessment data, as they intervene with follow-up initiatives. Such relationship between students’ self-reporting as assessment data and teachers’ intervention to enhance assessment data has rarely been reported in the existing literature.

The current study also has limitations. As mentioned earlier, the ADQ was not directly evaluated based on the actual accuracy of assessment data, but was indirectly based on teachers’ judgment about its accuracy. In other words, the ADQ may need to be more objectively assessed. Additionally, the within-group design on the IoI variable may also have brought about a threat to validity, because the participants, as they processed a later scenario, might have been unavoidably influenced by previously displayed scenarios.

## 5. Conclusions

This study aimed to explore how to enhance interactive self-reporting as a behavioural cue elicitor for online-classroom assessment. Specifically, the study adopted a large-scale questionnaire survey to investigate the effects of two independent variables, i.e., the intensity of interaction in interactive self-reporting and teachers’ professional experience, on the quality of assessment data in online teaching. Results indicated that the intensity of interaction has a significant impact on the quality of assessment data, while neither teachers’ professional experience nor the interaction between the two independent variables showed significant impacts.

This study carries some pedagogical implications. For instance, teachers may need to incorporate in their instructional design an interaction component as a follow-up step to further validate and enhance students’ self-reporting as an important source of assessment data. Moreover, the follow-up interaction needs to be regulated to balance the enhanced accuracy of assessment data and the compromised efficiency in conducting such assessment well. This is precisely where more empirical studies are needed for future research that further investigates the effects of the more crucial variables on the quality of the assessment data obtained via interactive self-reporting.

## Figures and Tables

**Table 1:** Mean scores for accuracy and efficiency ratings and the ADQ scores.

		Novice		Experienced		Total	
		*M*	*SD*	*M*	*SD*	*M*	*SD*
No interaction	Accuracy	3.58	1.203	3.43	1.176	3.50	1.191
	Efficiency	4.82	1.365	4.91	1.395	4.87	1.379
	ADQ Score	4.20	0.918	4.17	0.924	4.18	0.920
Brief interaction	Accuracy	4.57	1.608	4.49	1.643	4.53	1.624
	Efficiency	4.28	1.583	4.38	1.597	4.33	1.589
	ADQ Score	4.42	1.089	4.44	1.133	4.43	1.110
Elaborate interaction	Accuracy	4.83	1.330	4.80	1.392	4.82	1.360
	Efficiency	3.66	1.269	3.37	1.233	3.52	1.258
	ADQ Score	4.24	0.914	4.09	0.941	4.17	0.930

## Data Availability

A publicly available dataset was analysed in this study. This data can be found here: https://pan.baidu.com/s/1iMrpNk607gp_KQO-w2NeXg (accessed date 11 June 2022) (password: data).
